# The Use of Human Epididymis 4 and Cancer Antigen 125 Tumor Markers in the Benign or Malignant Differential Diagnosis of Pelvic or Adnexal Masses

**DOI:** 10.4274/balkanmedj.2016.0223

**Published:** 2017-03-28

**Authors:** Zehra Nihal Dolgun, Canan Kabaca, Ateş Karateke, Cem İyibozkurt, Cihan İnan, Ahmet Salih Altıntaş, Cihan Karadağ

**Affiliations:** 1 Clinic of Obstetrics and Gynecology, Zeynep Kamil Women and Children’s Training and Research Hospital, İstanbul, Turkey; 2 Department of Obstetrics and Gynecology, İstanbul University İstanbul School of Medicine, İstanbul, Turkey; 3 Department of Obstetrics and Gynecology, Trakya University School of Medicine, Edirne, Turkey; 4 Department of Obstetrics and Gynecology, Marmara University Training and Research Hospital, İstanbul, Turkey

**Keywords:** Tumour marker, ovarian cancer, adnexal mass, human epididymis 4, cancer antigen 125

## Abstract

**Background:**

Ovarian cancer is one of the highest mortality cancers in gynaecology. Discrimination of benign masses from malignant ones may sometimes become a challenge for the clinician since there is not a reliable tumour marker, thus some unnecessary, highly morbid operations can be performed.

**Aims:**

To explore the efficacy of human epididymis 4 (HE 4) and cancer antigen 125 (CA 125) markers in differentiating malignant and benign pelvic masses of ovarian origin and to identify the cut-off points for those markers.

**Study Design:**

Prospective study.

**Methods:**

Fifty-one patients who were diagnosed and planned to undergo surgery for ovarian mass between June 2008 and December 2008 were enrolled into this study. Preoperative venous blood samples were taken and frozen for marker investigation and final diagnoses were concluded by histopathological examination. After recruitment of all cases CA 125 and HE 4 levels were evaluated.

**Results:**

The statistical analysis did not indicate any statistically significant difference between the CA 125 levels of the patients with malignant and benign adnexal masses (p=0.105). The HE 4 levels of the patients with malignant adnexal masses were higher at a statistically significant level compared to the patients with benign adnexal masses (p=0.002). For HE 4 tumour marker and at the cut-off point of >25 pM, sensitivity was 1, specificity 0.40, positive cut-off value 0.19, negative cut-off value 1, accuracy 0.47 and positive likelihood ratio 1.65.

**Conclusion:**

Human epididymis 4 is a better diagnostic tool than CA 125 in benign-malignant discrimination of adnexal masses. The cut-off value of 25 pmol/L for human epididymis 4 will contribute to providing proper guidance to patients with adnexal masses and applying the proper treatment method.

Adnexal mass is the common name for tumours originating from fallopian tubes, ovaries and broad ligament due to functional, congenital, inflammatory and neoplastic processes. Most of them are of ovarian origin and benign in character (1) while approximately 23% of gynaecological cancers are of ovarian origin. Nearly 1 in 70 woman will suffer from ovarian cancer during their lifetime with a mortality rate of 47% among female genital system cancers (2). The majority of ovarian cancers are of epithelial origin and roughly two-thirds of epithelial ovarian cancers are diagnosed at stage 3 or stage 4 (3). Cancer-related abdominal pain and distension, menstrual irregularity, dyspepsia and other digestive system symptoms start to present only at the advanced stages (3,4). Early diagnosis is very important for decreasing mortality, performing satisfying surgery, increasing the patient’s quality of life, and minimizing treatment costs in ovarian cancer. Therefore, predicting whether the adnexal mass has a high risk of ovarian malignancy in the preoperative period is very important for determining the operative procedure to be performed, informing the patient properly and preventing any unnecessary procedures.

The confusion about the nature of the masses is mostly due to the lack of a convenient tumour marker. Until recently the only discriminator was the cancer antigen 125 (CA 125) tumour marker. But this marker is known to be elevated not only in cancer but also in any situation resulting with peritoneal irritation, such as tuberculosis, and even in the menstruel cycle (5).

Human epididymis 4 protein (HE4) is a novel tumour marker, which is claimed to differentiate malignant ovarian tumours from the benign ones. Recent studies show close correlation of HE4 with epithelial ovarian cancers in particular (6). But the cut-off point to detect the difference of the nature of the mass remains unclear.

In our study, we aimed to examine the value of CA 125 and HE4 in terms of pelvic mass differential diagnosis and also to find a cut-off point for HE 4.

## MATERIALS AND METHODS

After the approval of this prospective study by the Ethics Council of Zeynep Kamil Women and Childrens' Training and Research Hospital with the approval number 6833\44, written consents were received from all the individuals. Fifty-one patients who were seen in the outpatient clinic and diagnosed as having pelvic or adnexal mass and scheduled for surgery were enrolled in the study. Venous blood samples were taken preoperatively to examine CA 125 and HE4 tumour markers. These blood samples were centrifuged for 10 minutes at 3500 rpm, and then serums were separated and kept frozen at -80 °C until the tumour markers were studied. All the operations were performed at the Gynaecological Diseases Endoscopic Surgery and Gynaecological Oncology clinics of Zeynep Kamil Women and Childrens' Training and Research Hospital between June 2008 and December 2008. In addition to removing the masses, full surgical staging and debulking surgery was performed in patients whose results were malignant according to frozen pathologic examinations.

On the testing day, the serums taken for CA 125 and HE4 were brought to room temperature, and then 0.1 mL of standard, patient serum or control serum of the kit were put into separate proper test tubes. CanAg HE4 kits (Fujirebio Diagnostics) were used for HE4. SEAC Brio machine was used to run the test with the ELISA method (Pendik Laboratory) by incubating at 25 °C, and the results were read at 620 nm. CA 125 ‘was studied automatically with the ELISA method in an Immulite 2000 (Immulite 2000, Siemens, Erlangen, Germany) machine with the ELISA method automatically. The results were compared with standard curves, and CA 125 and HE4 levels were found.

The operation specimens of patients were studied at the Pathology Clinic of Zeynep Kamil Women and Children's Training and Research Hospital and classified as histologically benign or malignant. According to that, 45 masses were benign and 6 were malignant. The surgical staging of the patients diagnosed with malignancy was recorded along with the pathology results. Surgeons and pathologists performed the operations and histopathological diagnostic evaluations without knowing the results of the HE4 tumour marker. CA 125 and HE4 results were evaluated with histopathological diagnoses.

### Statistical analyses

In this study, the statistical analyses were conducted with NCSS Statistical Software 2007 (NCSS LLC, Utah, USA) package program. Descriptive statistical methods (median, standard deviation, mean) were used for data evaluation; Mann-Whitney U test was used for comparing paired groups; and chi-square test was used to compare qualitative data. Sensitivity, specificity, positive cut-off value, negative cut-off value, accuracy, and positive and negative likelihood ratio (LR) values were calculated for the variables. Logistic regression was applied for cancer existence. Area under curve (AUC) was calculated for CA 125 and HE4 according to variables. Results were evaluated with a significance level of p<0.05 and a confidence interval of 95%.

## RESULTS

A total of 51 patients were included in the study. We found that 45 of them had benign and 6 of them had malignant histopathologic results. One patient had a borderline serous tumour, and one patient had a borderline mucinous tumour. Those two patients with borderline results were included in the benign group. The average age of the patients with benign adnexal masses was significantly lower than the average age of patients with malignant adnexal masses (p=0.014) (Table 1).

The most frequent benign pathologies were benign serous cystadenoma (33.3%) and endometrioma (26.6%). These two histopathological diagnoses constituted 60% of all benign pathologies (Table 2). The distribution of malignant histopathological diagnoses is shown in Table 2. Both granulosa cell tumours were stage 1A, and carcinosarcoma was stage 3A. Stage could not be determined for leiomyosarcoma. For the two patients who had endometrioid adenocancer, one of them was stage 1A, and the other was stage 3C.

The averages and distribution of CA 125 levels according to the histopathological diagnoses are shown in Table 3. Among those patients with benign histopathology, the patients with the highest CA 125 levels were the ones with abscess and fibrothecoma. Average value was 176.75±149.53 U/mL for abscess and 181.5±244.27 U/mL for fibrothecoma. Among malignant diagnoses, the highest values were seen in epithelial ovarian carcinoma (endometrioid adenocarcinoma) (1427.5±1983.44 U/mL). CA 125 value was 225 U/mL for carcinosarcoma and 20 U/mL for leiomyosarcoma. CA 125 value was 191 U/mL for borderline serous tumour and 9 U/mL for borderline mucinous tumour. The statistical analysis did not indicate any statistically significant difference between the CA 125 levels of the patients with malignant and benign adnexal masses (p=0.105) (Table 4).

The distribution of HE4 levels according to the histopathological diagnoses is shown in Table 3. Among the histopathology results of benign adnexal masses, the highest value was an average of 33.75 pM with fibrothecoma. While HE4 value was 40 pM in borderline serous tumour, it was 36 pM in borderline mucinous tumour. Among the diagnoses of malignant adnexal masses, the highest values were seen in epithelial ovarian cancers. The average HE4 value was 159±9.9 pM in endometrioid adenocancer histology. The average HE4 value was 36±14.14 pM in granulose cell tumours, while HE4 value was 56 pM for carcinosarcoma and 86 pM for leiomyosarcoma. The HE4 levels of the patients with malignant adnexal masses were higher at a statistically significant level compared to the patients with benign adnexal masses (p=0.002) (Table 4).

The logistic regression analysis, which we performed to find the efficacy of CA 125 and HE4 in differentiating groups with malignant and benign adnexal masses, the CA 125 tumour marker was statistically insignificant in differentiating between benign and malignant adnexal masses (p=0.491), whereas HE4 was found to be statistically significant in this differentiation (p=0.025). We found that HE4 was affected by cancer existence at a statistically significant level but the β coefficient was 1.11 (Table 5).

Various values of sensitivity, specificity, positive cut-off value, negative cut-off value, accuracy, and positive and negative LR values were calculated with the purpose of determining a cut-off point for HE4 tumour marker. Receiver operating characteristic (ROC) curve indicated that the cut-off point for the malignant adnexal mass group was 25-30 pM. For HE4 tumour marker and at the cut-off point of >25 pM: sensitivity was 1, specificity 0.40, positive cut-off value 0.19, negative cut-off value 1, accuracy 0.47 and LR (+) 1.65 (Table 6, 7).

**Figure 1:**
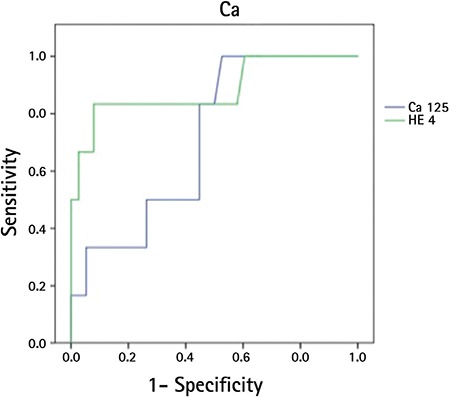
ROC curves for patients with malignant adnexal masses

For CA 125 tumour marker and at the cut-off point of >35 U/mL: sensitivity was 0.50, specificity 0.59, positive cut-off value 0.16, negative cut-off value 0.88, accuracy 0.58 and positive LR (+) 1.22 (Table 7). ROC curve arched with CA 125 and HE4 markers for patients with malignant adnexal masses indicated that the AUC of HE4 was significantly higher than the AUC of CA 125 (Table 8, Figure 1).

## DISCUSSION

Differentiating malignant and benign adnexal masses in the preoperative period remains an unsolved problem today. Because of the the lack of the histopathological diagnosis is often made during or after the operation. Ultimately, this situation can result in inadequate surgeries, improper treatments, and at times it leads to unnecessary and costly procedures. While ultrasound can detect pelvic masses successfully, it has a low specificity in differentiating whether a mass is benign or malignant. Although specificity increases with Doppler ultrasonography, this technique’s performance varies considerably according to the person performing it (7,8). The most frequently used marker for predicting ovarian cancer in pelvic masses is CA 125. However, the predictive power of this marker is inadequate. Its specificity is limited by the fact that its serum concentration increases with any peritoneal irritation, particularly in benign lesions such as endometriosis and fibroids (9).

CA 125 was shown in fallopian tubes, endocervix, endometrium, pericardial cells periton and pleura with mesothelial origin. After Bast et al. (10) discovered CA 125 in 1981 and reported that serum levels were over 35 U/mL in 82% of cases where ovarian cancer diagnosis was surgically finalized, many studies were conducted on this tumour marker. The subsequent studies reported the existence of CA 125 in kidney, lung, stomach, gall bladder, pancreas, colon and even in healthy adult ovaries (most recent studies), and this shows us that CA 125 is not specific to the ovary and that it has a broad distribution in human tissues (11). It is well known that its level increases in many diseases, particularly in premenopausal women such as endometriosis, myoma, salpingitis, and benign ovarian cyst. The literature reports that less than 4% of the patients who had abnormal CA 125 levels were diagnosed with ovarian cancer, and that CA 125 did not have an adequate positive predictive value for the screening of ovarian cancer (7,12).

In our study, when the upper limit for preoperative serum CA 125 level was taken as 35 U/mL, there was no significant difference between the preoperative CA 125 levels of the patients with malignant and benign adnexal masses. The literature reports that a method must have a minimum 10% positive cut-off value so that it can be used as a screening test for ovarian cancer (13). In our study, we detected a 16% positive cut-off value, which was consistent with the literature. However, since the sensitivity and specificity ratios we detected were low, we concluded that CA 125 measurements were not suitable for general screening or as a preoperative marker of the malignant-benign differentiation. In our study, the benign cases were more than the malignant cases, and this might have caused the low positive predictive value. We found that among benign masses the CA 125 levels in fibrothecoma and abscess formation were higher than the other benign masses.

HE4 is a molecule with protein structure. Reports state that it is secreted more in epithelial ovarian cancers. HE4 is essentially secreted from the reproductive system and upper airways, and it can be detected in blood serum (6,14). Unlike CA 125, HE4 is not affected by many frequent benign gynaecological and medical conditions (6). The study by Hellström et al. (6) indicated that HE4 tumour marker was more significant than the CA 125 marker in differentiating between malignant and benign diseases (6). Moore et al. (15) found the serum levels of HE4 tumour marker statistically significant in differentiating between benign and malignant adnexal masses (15). Another study by the same researcher analysed a series of molecules, such as Her2, CA 72-4, activin, inhibin, HE4 in terms of the differential diagnosis of malignant and benign adnexal masses, and found that, among those molecules, HE4 had the highest sensitivity and specificity both by itself and also combined with CA 125. An even more interesting finding was that adding CA 125 to HE4 for detecting stage 1 tumour decreased sensitivity (16).

The studies focusing on the HE4 tumour marker reported that the molecule was not affected by the benign gynaecological and medical causes that increase the serum level of CA 125 and that there was no increase in serum level in those conditions (6). In our study, serum HE4 levels of the patients with malignant adnexal masses were significantly higher than the patients with benign adnexal masses, parallel to reports in the literature (15,16). The study by Piovano et al. (17) found that HE4 was superior to CA 125 with its high specificity and sensitivity in detecting ovarian cancer recurrences. The study by Chung et al. (18) found that HE4 was an important diagnostic marker in diagnosing ovarian cancer. Serum HE4 levels were significantly higher in ovarian cancers compared to the other benign ovarian tumours. This indicated that the sensitivity of HE4 was higher than CA 125 in diagnosing ovarian cancer. It was found that, particularly in the benign-malignant differentiation, HE4 had a lower false positive ratio (18).

In our study, in the logistic regression analysis to define the efficacy of CA 125 and HE4 markers in differentiating malignant and benign adnexal masses, the CA 125 tumour marker was found to be statistically insignificant, whereas HE4 was found to be statistically significant in this differentiation. Our findings are also consistent with the literature (5,6,11). In our study, ROC arches with CA 125 and HE4 markers for patients with malignant adnexal masses indicated that the AUC of HE4 was significantly higher than the AUC of CA 125. This finding can be considered as an indicator of HE4’s predictive value and its superiority to CA 125.

The literature reports various cut-off values for the malignant-benign differentiation of HE4 serum levels (18,19). The study by Chung et al. (18) found that when the cut-off value for HE4 was taken as76 pM, the sensitivity was 78.1% and specificity was 86.8%. In the same study, the cut-off value for CA-125 was taken as 37.45 U/mL, and at this value, sensitivity was 84.4%, and specificity was 67.4%. In the study by Michalak et al. (19), the cut-off value for HE4 was taken as 140 pM for differentiating between malignant and benign masses, and they found that sensitivity was 85.2% and specificity was 94.6%. When the cut-off value was taken as 74 pM in the same study, sensitivity and specificities were 88.9% and 85.7%, respectively (19). In our study, we looked for a cut-off value for HE4 serum levels. We studied sensitivity, specificity, positive cut-off value, negative cut-off value and LRs in various cut-off values. We tried to find a cut-off value by arching the ROC curve of these parameters. The breaking point of the ROC curve was between 25 and 30 pM. At the cut-off point of >25 pM: sensitivity was 1, specificity 0.40, positive cut-off value 1, and positive LR was 1.65. At the cut-off point of >30 pM, we found: sensitivity 0.83, specificity 0.49, positive cut-off value 1.19, and positive LR 1.63. At the cut-off point of >25 pM, the fact that positive LR was 1.65 and negative LR was close to 0.00 made us think that this cut-off point was more appropriate. However, there is need for further and broader studies to ascertain its accuracy and acceptability.

## CONCLUSION

Our study informed that the HE4 tumour marker was significantly superior to the CA 125 tumour marker in differentiating between malignant and benign adnexal masses. Considering that the early and accurate diagnosis of ovarian cancer has a crucial role in directing patients to the tertiary centres where they can get the right treatment and be operated on and followed by gynaecologist oncologists, the predictive value of HE4 is a guiding light. Having the right treatment for the right patient will optimize the burden to the country budget, and also anxiety and uncertainty will decrease as the patient and patient’s family can be informed correctly. At this point, the results of our study support the fact that HE4 tumour marker is specific to ovarian malignancies, and that it is revealing in the early stage.

## Figures and Tables

**Table 1:**
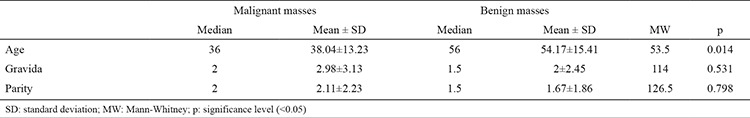
Descriptive characteristics of patients

**Table 2:**
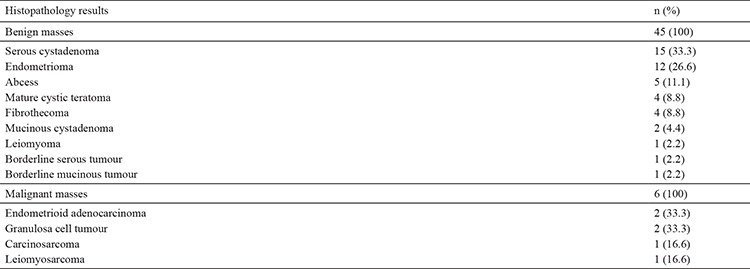
Classification of the adnexal masses

**Table 3:**
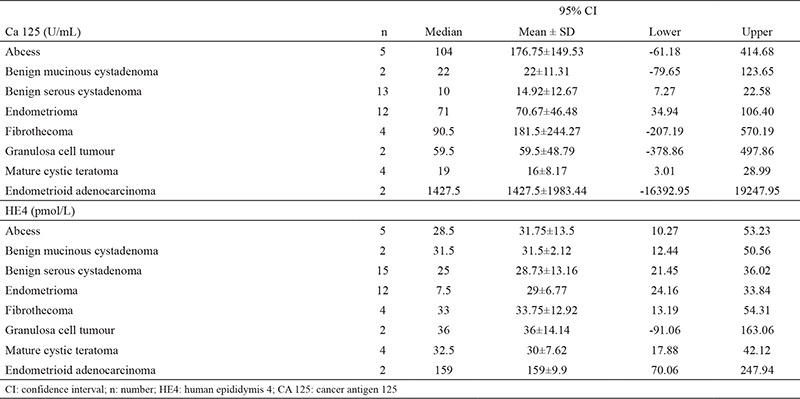
Marker levels of the adnexal masses

**Table 4:**
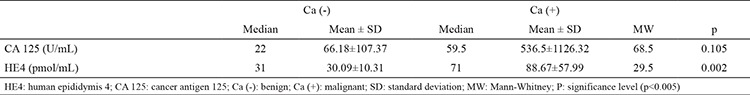
Marker values in differentiating benign and malignant cases

**Table 5:**
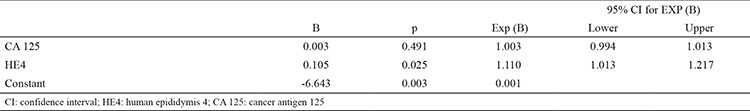
Impacts of CA 125 and HE4 in differentiating adnexal masses

**Table 6:**
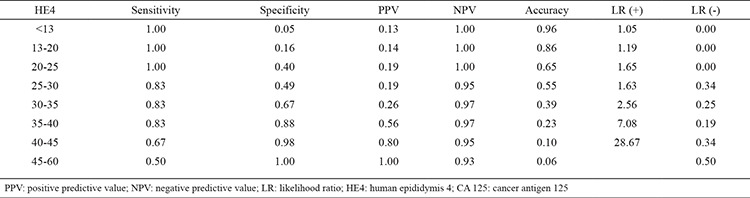
HE4 cut-off value

**Table 7:**
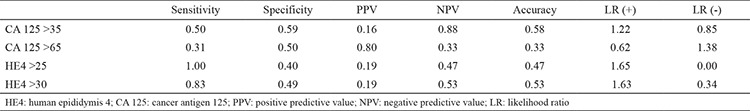
Borderline values for CA 125 and HE4

**Table 8:**
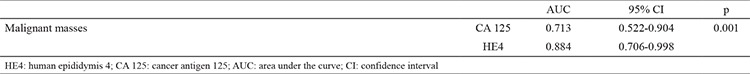
Area under receiver operating characteristic curve for patients with malignant adnexal masses
